# Effects of fatigue induced by repeated-sprint on kicking accuracy and velocity in female soccer players

**DOI:** 10.1371/journal.pone.0227214

**Published:** 2020-01-07

**Authors:** Víctor Torreblanca-Martínez, Fabio Nevado-Garrosa, Fernando M. Otero-Saborido, José A. Gonzalez-Jurado

**Affiliations:** 1 Faculty of Sport Sciences, Pablo de Olavide University, Seville, Spain; 2 Department of Physical Education, Sport and Human Movement, Autonomous University of Madrid, Madrid, Spain; Universidade Federal de Juiz de Fora, BRAZIL

## Abstract

The aim of this study was to investigate the effects of fatigue induced by repeated sprint in the kicking accuracy and velocity in female soccer players. Eighteen Under-23 female soccer players from a Spanish professional club were subjected to a fatigue protocol based on a repeated-sprint ability (RSA) test. Measurements of the kicking velocity (maximal ball velocity) and accuracy (Loughborough Soccer Shooting Test) were taken before and after fatigue induction. Correlations between the change in the maximal ball velocity/accuracy and the heart rate (HR), the fatigue index (FI), the sprint decrement (S_dec_) and the rating of perceived exertion (RPE) were made. There was a significant difference between maximal ball velocity under fatigue conditions with respect to non-fatigue conditions (p = 0.001; ES = 0.89). However, despite a lower kicking accuracy punctuation with fatigue, this was not statistically significant (*p* = 0.433; ES = 0.22). Significant correlations were found between the maximal kicking velocity and the FI (*r* = 0.632, *p* < 0.01) and the S_dec_ (*r* = -0.554, *p* < 0.05) and between the kicking accuracy and the RPE (*r* = -0.506, *p* < 0.05). In conclusion, there was a significant reduction in the maximal kicking velocity, but not in the kicking accuracy, under fatigued conditions. The RSA-related FI and S_dec_ were the best predictors of the maximal kicking velocity and the RPE for the kicking accuracy.

## Introduction

Kicking is the most widely studied soccer skill [1], as it is the defining action in the game [2], with 80.69% of goals being scored by means of this action [1]. A development of fatigue may be observed, with a reduction of the high intensity activities, during and toward the end of the game owing to major metabolic changes [3,4].

Maximal ball velocity and accuracy have been the most studied variables related to the kicking action, with studies on the effects of diverse exercise protocols in both factors [5], such as match simulations [5], circuits [6–9], running on a treadmill at different intensities [10,11], incremental tests [12], squats [13], countermovement jumps [14], knee flexion and extension [15] and the real competition [16]. Therefore, a large number of fatigue protocols are found, which makes it difficult to interpret the results of this concerning the kick and the capacity to generate fatigue; in some cases, the physiological demands of the game are not faithfully reproduced [5].

We found different measurements to ensure the presence of fatigue when the player kicks under fatigue conditions in the previously described protocols, such as the heart rate [5–10,12,16], the rate of perceived exertion (RPE) [5,7,12,16], or the blood lactate [5,7,8,12].

Some studies have reported results about the effect of fatigue in kicking accuracy [5,9,12,13]; however, all of them used only male players and no report has been found to study this in female soccer players. In this case, the studies achieved the same conclusion: fatigue affects kicking accuracy. Only one study has reported data about correlations between fatigue variables and a decrement in kicking accuracy, showing a decrement in kicking accuracy close to the second lactate threshold [12].

Many studies about the effect of fatigue on kicking velocity were found [6–8,10–12,14–16], but only one included female soccer players in the sample [11]. We found a disparity of results related to kicking velocity, some studies concluding that fatigue affects kicking velocity [6–8,11,12,15] and, in other cases, that fatigue does not affect kicking velocity [10,14,16]. In addition, a decrement in kicking velocity was correlated with the increment in a shuttle-running test from the second lactate threshold [12]. Yet, a decrement in a fatigue protocol based on countermovement jumps did not establish correlations with the decrement in the maximal kicking velocity [14].

According to this, a large diversity of results has been reported and many different methodologies have been applied, related to the induction of fatigue and its effects on soccer kick efficacy. In addition, few studies including female soccer players are found, being known the kinematic differences reported in kicking between male and female soccer players [17,18]. So, the aim of this research was to investigate the effects of fatigue induced by repeated sprint in kicking accuracy and velocity in female soccer players. It was hypothesized that fatigue affects kicking accuracy and velocity.

## Methods

### Participants

A convenience sample of 18 U-23 female soccer players was recruited, with the following characteristics: age (Mean ± SD, years) = 18.44 ± 1.75; range = 17–23; body mass (Mean ± SD, kg) = 56.08 ± 6.50; height (Mean ± SD, cm) = 161.61 ± 4.90.

Players belonged to the second team of a professional Spanish club. This team has competed in the second national female soccer league for the last 9 seasons. Three weekly training sessions are performed during the competitive period. At the time of the study, no injuries were diagnosed and no player was in a recovery process.

All participants and their parents or guardians were informed in advance about the purpose of the study and the type of evidence to be submitted. Each of the players and their parents or guardians gave their signed informed assent following the recommendations of the Declaration of Helsinki. The study was approved by the Institutional Review Board of Pablo de Olavide University.

### Instruments

Anthropometric measures were taken according to standard procedures [19]. The body mass was measured with a reliable weighing scale Seca^®^ 869 (Seca GmbH & Co, Hamburg, Germany) and the height with a stadiometer (Soehnle^®^ 5003, China).

The repeated-sprint ability test (RSA) reproduces maximal or near maximal effort with brief recovery intervals in team sports [20]. RSA performance was determined by using two Microgate Witty Gate photocells (Microgate®, Italy), with a Microgate Witty Timer receptor. The distance of 12x30 m all-out running sprint, with 30 seconds of recovery, has previously shown the capacity to induce evident fatigue conditions [21]. In order to know the degree of fatigue achieved before the second kick, the Fatigue Index (FI) and the Percentage decrement score (S_dec_) have been proposed for determining the manifestation of fatigue [20]:

$$FI=100x\frac{\left(Sbest-Sworst\right)}{Sbest}$$

$$Sdec(\%)=\left(1-\frac{S1+S2+S3+\cdots +Sfinal}{Sbestxnumberofsprints}\right)x100$$

The heart rate was monitored during the entire protocol. All the players wore a Polar heart rate monitor Polar V800 (Polar^®^, Finland). The rating of perceived exertion (RPE) measured by the Borg Scale [22] was recorded at the start and at the end of the RSA test.

The maximal kicking velocity has been a common measurement in different studies that aimed to determine kicking velocity, which was obtained using a radar gun [7,23]. In the present study, a Stalker ATS II radar (Stalker^®^, USA) was used. It has a precision time of 0.01 seconds, a velocity range from 1–1432.3 km/h and the capacity to detect ball movement over a distance of 152.40 meters. The kicks took place from the penalty spot with the dominant leg.

The kicking accuracy was measured with the Loughborough Soccer Shooting Test (LSST), previously validated [24] and conducted in different studies that aimed to determine kicking accuracy in soccer [9,25,26]. The kicking zone was marked at 16.5 m from the goal. The shooting area was 8.5 x 8.5 m, marked by four traffic cones and a wooden bench. The full goal was split into scoring zones with a 7-cm reflective red and white tape. The targets were given point values that reflected better shots and chances of scoring in a competitive soccer match [25] (5 points for kicks to the corner of the goal, 3 points for kicks near the goal post, 2 points for kicks close to the center and 1 point for kicks to the center). Before each kick, the players had to sprint to one of the four cones, come back to the center, pass a ball to the bench, control the rebound and kick. In order to avoid a possible decrement in fatigue conditions, as has been reported in previous studies [11], each kick was done with only 6 seconds of recovery, in the same way as has been carried out in some protocols similar to the LSST [12]. The kicks were recorded with two Sony Handycam DCR-SR35 cameras (Sony^®^, Japan).

### Testing procedures

Testing was conducted over three different days at the Rayo Vallecano de Madrid S.A.D. sport center on an artificial grass pitch, where the team usually trains and plays league matches, under stable weather conditions. All the participants were equipped with their own soccer boots and regular training outfit. Ten Adidas Errejota RFEF approved balls were used (Adidas^®^, Germany).

**Fig 1:**
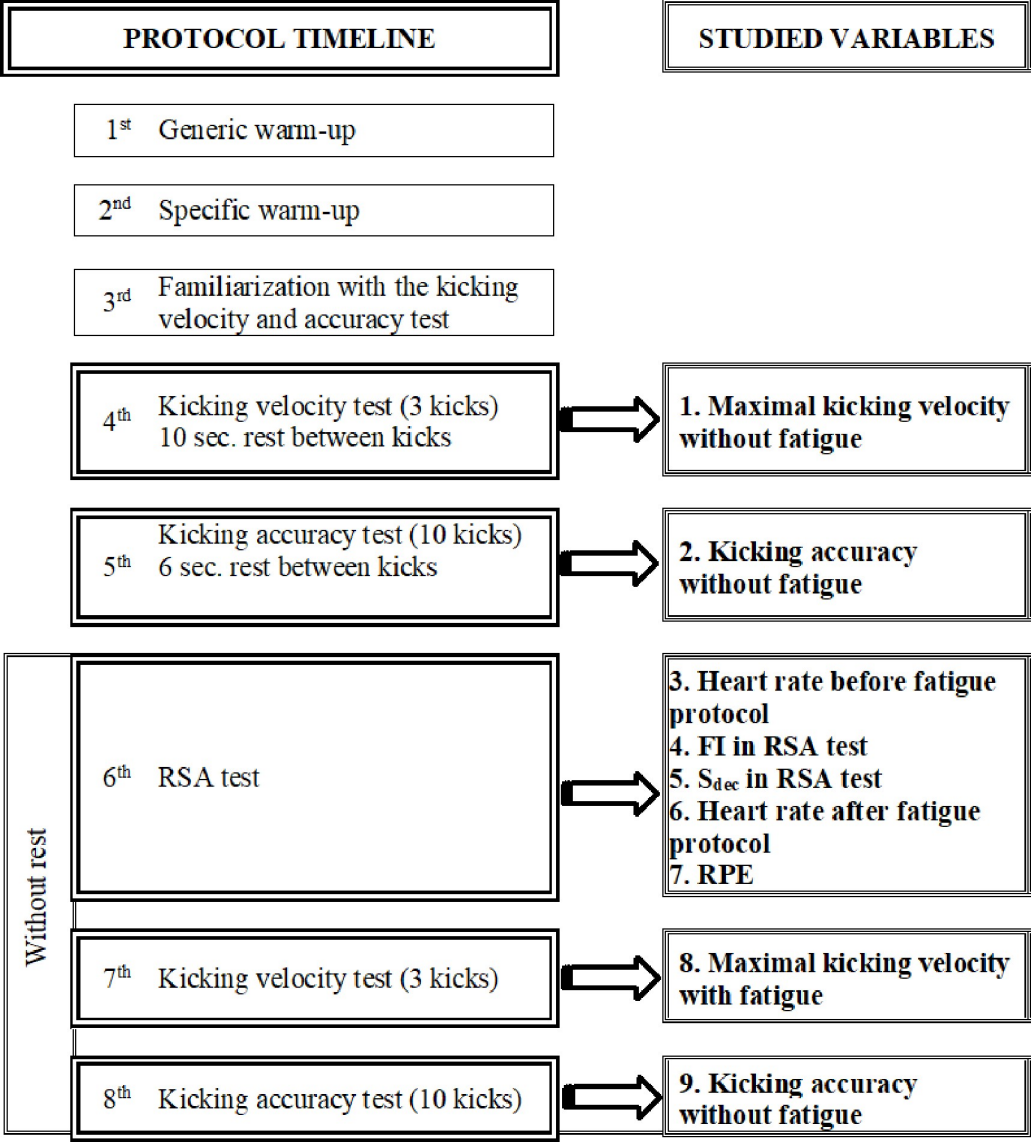
Timetable and variables flowchart.

The research protocol was (Fig 1):

1. Generic warm-up (8 minutes): articular mobility, cardiovascular activation (jogging) and stretching.
2. Specific warm-up (4 minutes): kicking and sprint.
3. Familiarization with the kicking velocity and accuracy test.
4. Three maximal instep kicks with ten seconds of recovery between each kick. The fastest was selected.
5. Ten kicks, following the LSST protocol, with 6 seconds of recovery between each trial, in order to achieve the maximum score in each kick. The total score of the ten trials was selected.
6. An RSA test, which consisted in twelve 30-meter sprints, each separated by 30 seconds of passive rest.
7. Immediately after, three maximal instep kicks were performed. The fastest was selected.
8. Without a rest, the LSST protocol was repeated.

Variables studied (Fig 1):

1. Maximal kicking velocity without fatigue.
2. Kicking accuracy without fatigue.
3. Heart rate before the fatigue protocol.
4. FI in RSA test.
5. S_dec_ in RSA test.
6. Heart rate after the fatigue protocol.
7. RPE.
8. Maximal kicking velocity with fatigue.
9. Kicking accuracy with fatigue.

### Statistical analyses

For the statistical analyses, the software IBM SPSS statistics 22 was used.

Regarding descriptive statistics, the mean and the standard deviation were calculated.

The confidence level of the measurements was estimated at a 95% confidence interval for the mean.

In order to know the consistency of the measurements, the intraclass correlation coefficient (ICC) and the coefficient of variation (CV) were calculated between repeated kicking measurements. The two-way random absolute agreement model was used.

With respect to inferential statistics, a paired sample t-Test or Wilcoxon was conducted, according to the normality, calculated through the Shapiro-Wilk test. The Cohen’s *d* effect size was also calculated [27], considering values of *d* < 0.3 small, *d* = 0.3–0.5 as moderate, *d* = 0.5–0.7 as large, *d* = 0.7–0.9 as very large and *d* > 0.9 as extremely large [28].

To know if there were correlations between the differences in the kicking velocity and the accuracy before and after the fatigue protocol and the fatigue variables, Pearson´s *r* was calculated, setting the statistical significance at *p* < 0.05.

Multiple Linear Regression was carried out to determine the relative contributions of the different fatigue variables to the change in the kicking accuracy and velocity under fatigue conditions.

## Results

Table 1 shows a descriptive analysis of the participants characteristics. In addition, the fatigue variables are included. High fatigue values are achieved after the fatigue protocol, with an average in the final heart rate of 178.61 beats per minute, a score of 15.22 in the RPE Borg’s scale and a decrement in RSA performance, showing an FI in RSA of -12.52% and an S_dec_ in RSA of -6.53%.

**Table 1:** Descriptive analysis of the sample and fatigue variables.

	Mean ± SD	CV	CI (95%)
**Age (years)**	18.44 ± 1.75	9.49	17.57–19.31
**BM**	56.08 ± 6.5	11.59	52.85–59.32
**Height (cm)**	161.61 ± 4.9	3.03	159.17–164.04
**Final HR (bpm)**	178.61 ± 11.84	6.63	172.71–184.50
**RPE**	15.22 ± 2.53	16.62	13.96–16.48
**FI RSA (%)**	-12.50 ± 4.8	-38.40	-14.89 - -10.11
**S**_**dec**_ **RSA (%)**	-6.53 ± 2.95	-45.18	-8.00 - -5.06

SD = Standard deviation; CV = Coefficient of Variation %; CI (95%) = 95% Confidence Interval; BM = Body Mass (kg); Final HR = Heart rate after fatigue protocol in beats per minute; RPE = Rate of perceived exertion in Borg’s scale; FI RSA = Fatigue index in repeated-sprint ability; S_dec_ RSA = Percentage decrement score in repeated-sprint ability.

**Fig 2:**
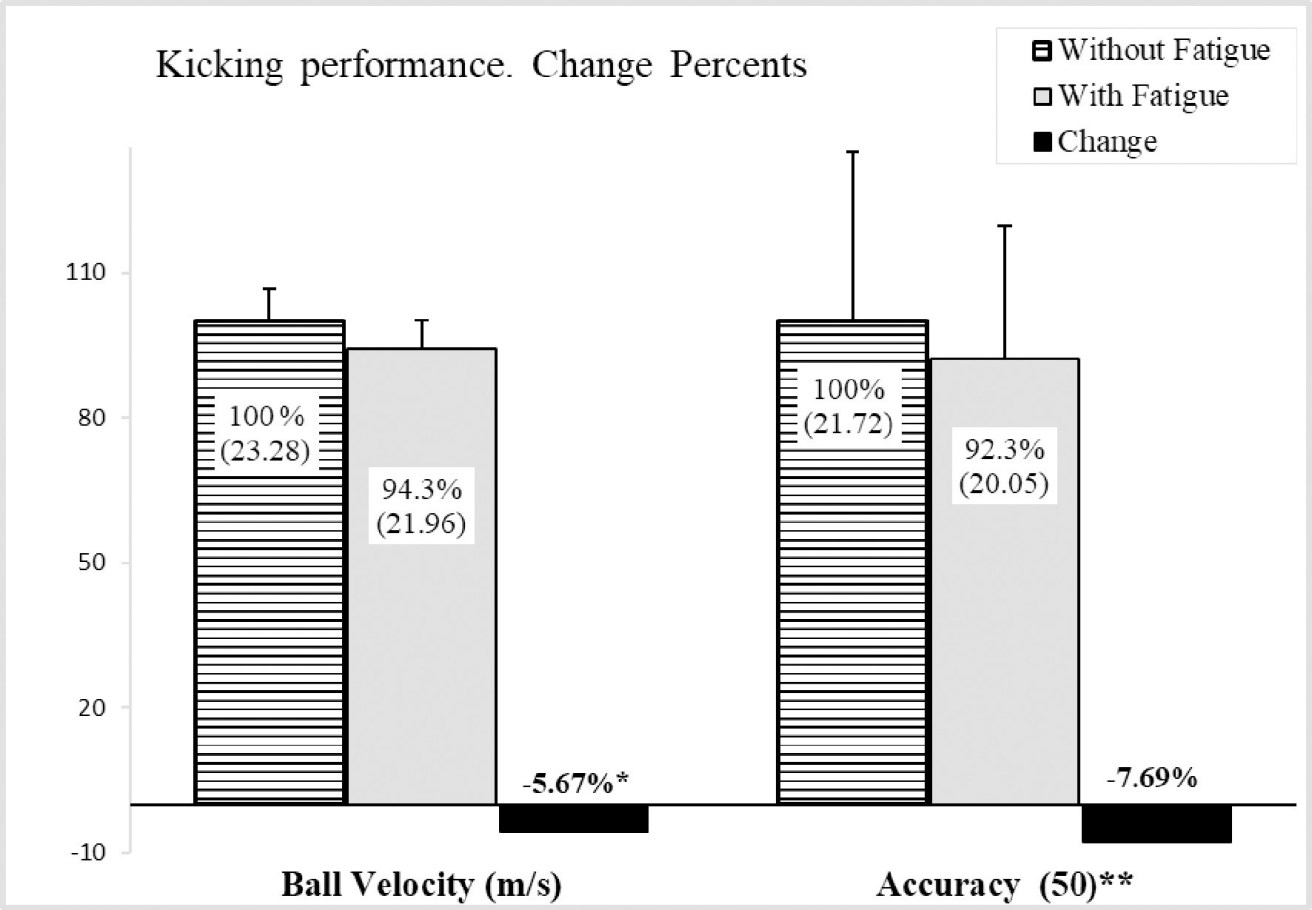
Kicking variables. **Percent Relative changes according to "without fatigue" values.** * significant differences (Paired sample t-test). ** maximum possible score in accuracy.

The highest CV of the first kick was 0.06 and 0.07 for the second kick. In addition, the first kick ICC was 0.871 and 0.875 for the second kick. Significant differences were observed in the kicking velocity between fatigue and non-fatigue conditions (*p* = 0.001) and the effect size (0.89) was very large. Regarding the kicking accuracy, no significant differences were found between both measurements (*p* = 0.433) and the effect size obtained (0.22) was considered small (Table 2). A reduction of 5.67% for kicking velocity and 7.69% for kicking accuracy was obtained (Fig 2).

**Table 2:** Ball velocity (m/s) and mean accuracy in fatigue and non-fatigued conditions (Mean±SD).

	Without Fatigue	With Fatigue	Change	CI (95%)	*p* value*	Effect size^§^	
						Value	Magnitude
**Velocity (m/s)**	23.28 ± 1.59	21.96 ± 1.29	1.32 ± 1.32	0.66–1.98	0.001	0.83	Very large
**Accuracy (total score)**	21.72 ± 7.62	20.05 ± 5.51	1.66 ± 8.79	-2.70–6.04	0.433	0.22	Small

* Calculated with T-test.

§ Calculated with Cohen's effect size (Cohen, 1988).

In reference to the correlation between fatigue measures and the kicking velocity and the accuracy, there was a correlation between the kicking velocity change and the FI (*r = -*0.632, *p* < .01) and the S_dec_ (*r = -*0.554, *p* < 0.05) and between the kicking accuracy change and the RPE (*r = -*0.506, *p* < 0.05). However, there was no correlation between the kicking velocity change and the heart rate difference (*r =* 0.235, *p* = 0.347), the RPE (*r =* 0.380, *p* = 0.119) or the change in the kicking accuracy (*r =* 0.045, *p* = 0.859). At the same time, there was no correlation between the kicking accuracy change and the heart rate difference (*r =* 0.190, *p* = 0.450), the FI (*r =* 0.346, *p* = 0.159), the S_dec_ (*r =* 0.174, *p* = 0.491) or the change in the kicking velocity (*r =* 0.045, *p* = 0.859) (Table 3).

**Table 3:** Correlations between fatigue measures and kicking velocity and accuracy*.

	Kicking velocity	Kicking accuracy
**Heart rate**	0.235 (0.347)	0.190 (0.450)
**Fatigue index RSA**	-0.632 (0.005)	0.346 (0.159)
**Sprint decrement RSA**	-0.554 (0.017)	0.174 (0.491)
**Rate of perceived exertion**	0.380 (0.119)	-0.506 (0.032)
**Kicking velocity**	1	0.045 (0.859)
**Kicking accuracy**	0.045 (0.859)	1

*Pearson’s Correlation Bivariate: *r* (*p* value).

Table 4 shows a significant linear regression model for the difference in the kicking velocity, which includes the variables Heart rate difference before and after the fatigue protocol, FI and S_dec_ in RSA. There were no significant differences in the relative contribution of each variable to the kicking velocity, as shown by the individual Beta coefficients.

**Table 4:** Fatigue predictors for kicking velocity. Linear Regression.

	*R*^*2*^ (Sig.)	Standardized Beta coefficient (Sig.)
**Heart rate difference**	0.46 (0.02)	0.26 (0.21)
**Fatigue index RSA**		-0.86 (0.08)
**Sprint decrement RSA**		0.26 (0.58)

Table 5 shows a significant lineal regression model for the differences in the kicking accuracy, which includes the variables RPE, FI and S_dec_ in RSA. There were significant differences in the relative contribution of the variables RPE and S_dec_ in RSA to the kicking velocity, as shown by the individual Beta coefficients.

**Table 5:** Fatigue predictors for kicking accuracy. Linear Regression.

	*R*^2^ (Sig.)	Standardized Beta coefficients (Sig.)
**Rate of perceived exertion**	0.44 (0.03)	-0.63 (0.02)
**Fatigue index RSA**		0.87 (0.07)
**Sprint decrement RSA**		-1.03 (0.04)

## Discussion

The aim of this research was to investigate the effects of fatigue induced by repeated sprints in kicking accuracy and velocity in female soccer players.

The fatigue protocol applied in this study was intense enough to generate a tangible fatigue in the participants (Table 1). A similar protocol has been used in a previous study, and its ability to induce real fatigue in soccer players has been demonstrated [21]. In addition, RSA reproduces the effort that would be expected in team sports [20], which has been the main problem in other protocols implemented to induce fatigue in previous studies [5]. Consequently, the participants performed the second tests of kicking velocity and accuracy under real fatigue conditions.

The maximal ball velocity before the fatigue protocol was 23.28 ± 1.59 m/s (Table 2). Similar results were observed in previous studies with elite female soccer players [29,30]. Regarding the kicking accuracy, there are no references to the LSST protocol in female soccer players, previous research under non-fatigue conditions carried out with semi-professional [9] and experienced [26] male soccer players reported similar results. According to the results obtained in the difference between the maximal kicking velocity before and after the fatigue protocol (Table 2), there is a significant reduction in the kicking velocity and a very large effect size (p = 0.001; ES = 0.89). These results are consistent with other studies with female players [11] and male players [6–8,11,12,15]. Furthermore, several studies carried out with male soccer players reported a decrease in the kicking velocity after the fatigue protocol; however, no statistical significance was found [10,14,16]. These differences could be due to the protocol used, which did not reproduce the game characteristics, such as running at 80% of the maximal heart rate [10] or countermovement jumps [14]. Another possible explanation, as has been reported in a previous study [11], is that the maximal kicking velocity is recovered to pre-fatigue levels just one minute after the end of the protocol. Therefore, performing the kicking test at the same time as the CMJ test [10] or carrying out the kicking test in the break time and at the end of the match [16] but not immediately after the fatigue protocol, could make it possible to recover the maximal kicking velocity to pre-fatigue levels.

On the other hand, the kicking accuracy before and after the RSA test registered a decrement in the total score when doing the LSST (Table 2), but this was not statistically significant, having a small effect size (*p* = 0.433; ES = 0.22). These results disagree with previous studies with male participants [5,9,12,13]. We consider that diverse reasons explain this discrepancy. First, there is the kinematic differences reported in kicking between male and female soccer players [17,18]. This makes it impossible to establish similar results in both genders. Secondly, there is the previously cited fast recovery after the fatigue protocol for kicking ability [11]. The LSST was carried out after the maximal velocity kicking test; thus, before doing the LSST, the players could have recovered similar values to those before the fatigue protocol. The third argument is related to the kicking accuracy test. Few studies have taken measurements of kicking accuracy and the effect of fatigue using previously validated tests, such as the LSST or the 365 soccer shooting test [12]. In order to improve the ecological validity of these tests [12], the players were instructed to kick far from the goalkeeper position, so a higher score was given near the corners of the goal, as reflected in the LSST. From our point of view, decision making is crucial in these tests, so the score not only reflects the kicking accuracy, it also shows the player’s ability to take a risk (for example, choosing to make all the kicks to the center ensures the scoring of points), in both situations (without and under fatigue conditions). Lastly, kicking accuracy percentage reduction is bigger than the values of kicking velocity (-7,69%), despite not obtaining significant values. Maybe, it could be explained by the standard deviation values obtained in the LSST (Fig 2).

Table 3 presents the correlations between the fatigue variables and the change in the kicking velocity and accuracy. The parameters related to the performance in the RSA test have the highest correlations with the kicking velocity change, *r* = -0.632 (*p* < 0.01) for the FI and r = -0.554 (*p* < 0.05) for the S_dec_. These results demonstrate that the RSA was an effective fatigue protocol due to the high correlations between the decrement of the performance in this test and the consequent decrement in performance in the maximal kicking velocity. Yet, there were no correlations between the change in heart rate after the fatigue protocol and the reduction in the maximal kicking velocity (*r =* 0.235, *p* = 0.347). The kicking velocity is determined by achieving a high angular and lineal velocity, and a high coordination in the different segments of the kicking leg [31]. Therefore, a central measurement of fatigue, such as the heart rate, may not be the best predictor of performance in an explosive action, for instance kicking. Consequently, this might explain the different results in the decrement of the kicking velocity after the fatigue protocol in different studies that have used heart rate as a fatigue measurement [6–8,10,12,16]. The RPE score had no significant correlations either with the reduction in the kicking velocity (*r =* 0.380, *p* = 0.119) or with the reduction in the kicking accuracy (*r* = 0.045, *p* = 0.859).

With respect to the change in the kicking accuracy and its correlations with fatigue measurements, we only found a significant correlation between the RPE score and the decrement in the total LSST score (*r = -*0.506, *p* < 0.05), but not with the FI (*r =* 0.346, *p* = 0.159), the S_dec_ (*r =* 0.174, *p* = 0.491) or the heart rate (*r =* 0.190, *p* = 0.450). Nor did the kicking velocity reduction show any correlation with the kicking accuracy. It has been previously reported that, when the emphasis of the kick was accuracy, the ball velocity was reduced as a consequence of lower segmental velocities in the kicking leg [32]. The fatigue indicators in RSA are: limitation in energy supply, metabolic by-product accumulation and failures to fully activate the contracting muscle [20]. Therefore, a non-maximal demand during accuracy kicking, unlike maximal velocity kicking, could explain that there were no correlations between the RSA equations and the reduction in the LSST score. Finally, the RPE had a significant correlation with the kicking accuracy but not with the kicking velocity. This disparity between both measures could explain the diversity of the results that we find in studies that used the RPE as a fatigue measurement [7,12,16].

In order to detect the influence of each of the fatigue variables on the change in the kicking accuracy and velocity, two linear regression models were carried out. Table 4 shows the Linear Regression of the kicking velocity change. The statistically significant regression model does not include the RPE, probably because it is a subjective variable and has little effect on the kicking velocity decrement. According to the Beta coefficients, the FI in the RSA is the most influential variable in the kicking velocity after fatigue. However, none of them significantly predicted way the loss of velocity in the kick. Likewise, it was observed that the increment in the HR-change after the fatigue protocol and the S_dec_ in the RSA showed similar results in the Beta coefficients.

Table 5 shows the Linear Regression of the kicking accuracy change. In this case, the statistically significant regression model includes the RPE, but not the HR-change. In addition, the Beta coefficients of the RPE and the S_dec_ in the RSA obtained a significant difference, with the RPE being the most influential variable. From our point of view, these results reinforce the previous argument about the influence of decision making on the LSST, since a subjective variable, such as the RPE, which has no influence on the Linear Regression model of the kicking velocity, is now the most influential variable for accuracy; a physiological variable, for instance the HR-change, must not be included.

On the other hand, the inclusion of all the variables related to the RSA in the Linear Regression models for kicking velocity and accuracy (considering that the FI is the most influential variable in the kicking velocity model and the S_dec_ registered significant values in the kicking accuracy model), reinforces the use of the RSA as an effective protocol to generate a tangible fatigue for studying kicking variables, unlike other protocols used in different studies.

As future lines of research, a new test for measuring kicking accuracy should be previously validated in order to avoid the influence of decision making on the LSST or similar tests that consider the corners of the goal as the zones with the highest score. Perhaps a test that determines the distance of each kick from the center of a specific target, similar to a bulls-eye, might be better for determining the effect of fatigue on kicking accuracy. In addition, it could be interesting determining what type of training is the best way for reducing the effect of fatigue in kicking performance.

The main limitation of the present study was the sample size, which could be increased to a higher number of players, teams or player levels. Likewise, blood sample measurements, including lactate levels, could be included as measurements of the fatigue protocol and for studying correlations with the consequent reduction in the kicking velocity and accuracy. In addition this study lacks a priori sample size power analysis, owing to the difficult access to professional athletes who agree to take part in the study.

## Conclusion

The results show that there is a significant reduction in the maximal kicking velocity after the induction of fatigue by means of an RSA test in female soccer players. There is also a reduction in the kicking accuracy punctuation after the fatigue protocol, but this is not statistically significant.

As a practical application, following these study results, it could be advisable to program training exercises focusing on improving kicking performance after fatigue situations, especially in kicking velocity, owing to the fact of being the most affected variable in the research. It is necessary to take into account that this is a case study with a sample of an only female team.
